# Scaling up nanoscale water-driven energy conversion into evaporation-driven engines and generators

**DOI:** 10.1038/ncomms8346

**Published:** 2015-06-16

**Authors:** Xi Chen, Davis Goodnight, Zhenghan Gao, Ahmet H. Cavusoglu, Nina Sabharwal, Michael DeLay, Adam Driks, Ozgur Sahin

**Affiliations:** 1 Department of Biological Sciences, Columbia University, New York 10027, New York, USA; 2 Department of Physics, Columbia University, New York 10027, New York, USA; 3 Department of Chemical Engineering, Columbia University, New York 10027, New York, USA; 4 Department of Biomedical Engineering, Columbia University, New York 10027, New York, USA; 5 Department of Microbiology and Immunology, Loyola University Chicago, Maywood, Illinois 60153, USA

## Abstract

Evaporation is a ubiquitous phenomenon in the natural environment and a dominant form of energy transfer in the Earth's climate. Engineered systems rarely, if ever, use evaporation as a source of energy, despite myriad examples of such adaptations in the biological world. Here, we report evaporation-driven engines that can power common tasks like locomotion and electricity generation. These engines start and run autonomously when placed at air–water interfaces. They generate rotary and piston-like linear motion using specially designed, biologically based artificial muscles responsive to moisture fluctuations. Using these engines, we demonstrate an electricity generator that rests on water while harvesting its evaporation to power a light source, and a miniature car (weighing 0.1 kg) that moves forward as the water in the car evaporates. Evaporation-driven engines may find applications in powering robotic systems, sensors, devices and machinery that function in the natural environment.

## Abstract (web summary)

Harvesting energy from evaporation is constrained by the limited transport kinetics of materials and the slowly changing humidity of the environment. Chen *et al*. follow hierarchical design strategies to overcome these problems and create engines that start and run when placed at air-water interfaces.

## Graphical abstract

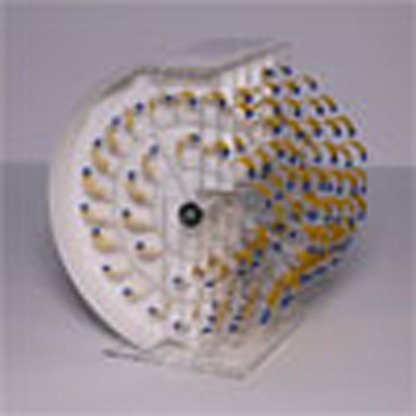

Despite the ubiquity of evaporation in the natural environment^1^ and the wide-ranging adaptations in the biological world that harness evaporation^2^,^3^,^4^,^5^, the potential of evaporation to power engineered systems is largely neglected. Nanoscale confinement of water in hygroscopic materials provides a means to convert energy from evaporation by generating mechanical force in response to changing relative humidity^6^,^7^,^8^,^9^,^10^,^11^,^12^,^13^,^14^,^15^,^16^,^17^,^18^,^19^,^20^,^21^. However, scaling up this phenomenon to create macroscopic devices faces multiple challenges: unfavourable scaling of hydration kinetics slows down actuation speeds at large dimensions; small strains complicate energy transfer to external systems; and, importantly, the slow rate of change of relative humidity in the environment limits the power output.

While evaporation carries a significant amount of energy^22^,^23^, it involves a slow rate of water transfer that limits the relative expansion and contraction of hygroscopic materials. Because the relative volume of the absorbed and released water is small, the pressure change generated during this process has to be large for efficient energy conversion. Water confined to nanoscale cavities within hygroscopic materials (Fig. 1a) can induce large pressures in response to changing relative humidity^24^,^25^,^26^; however, these nanostructures also limit the transport kinetics of water. Simply scaling up the dimensions of hygroscopic materials would not increase power, and may even lead to a decrease, because the time scale of wetting and drying typically depend on the square of the travel distance of water^19^.

Addressing the limitations in transport kinetics alone would not be sufficient to increase power, because in typical environmental conditions relative humidity changes on daily and seasonal timescales, which is too slow to generate practically useful levels of power. However, spatial gradients in relative humidity established near evaporating surfaces provide an opportunity. If a small portion of the power generated by the spores could be used to control the evaporation rate or, alternatively, move the spores in and out of the high humidity zone at the surface, the relative humidity experienced by the spores would change rapidly in a cyclical fashion.

Here, working with bacterial spores that exhibit strong hydration-driven actuation^20^, we present strategies to circumvent these challenges described above to create macroscale evaporation-driven engines. These engines start and run autonomously when placed at air–water interfaces, and operate as long as the air is not saturated. We demonstrate that these engines are able to power an electricity generator to light up LEDs and drive a miniature car as the water evaporates.

## Results

### Design strategies

**Figure 1:**
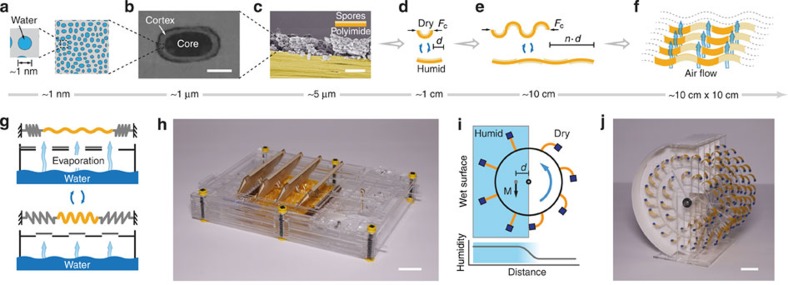
Scaling up hydration-driven nanoscale energy conversion. (**a**) Water confined to nanoscale cavities, conduits and surfaces within hygroscopic materials can induce large pressures in response to changing relative humidity. (**b**) A scanning electron microscopy image of the cross-section of a *B. subtilis* spore. Spores exhibit strong mechanical response to changing relative humidity^18^ by absorbing and releasing moisture. (**c**) A false-coloured s.e.m. picture of spores (grey) deposited on an 8-micrometre-thick polyimide tape (yellow). (**d**) The spore-coated films bend and straighten in response to changing relative humidity. (**e**) Patterning equally spaced spore layers on both sides of the plastic tape creates linearly expanding and contracting structures. (**f**) Stacking the tapes in **e** with air gaps between them results in a material that can be scaled in two dimensions without compromising hydration/dehydration kinetics. (**g**) A shutter mechanism can create oscillations. (**h**) Photo of a device that exhibit self-starting oscillatory movement when placed above water. (**i**) Rotary motion can lead to cyclical changes of relative humidity experienced by the spores. The increased curvature on the dry side shifts the centre of mass of the entire structure away from the axis of rotation and creates torque. (**j**) Photo of a device whose continuous rotation is powered by evaporation from the wet paper within the device.

We follow a hierarchical design strategy to enhance water transport kinetics in evaporation-driven macroscopic systems. We started with bacterial spores deposited on micrometre-thick plastic films (Fig. 1b,c). These films change curvature as a function of relative humidity (Fig. 1d). The overall movement can be made linear by coating alternating sides of longer tapes with spores (Fig. 1e). Assembling several tapes as a stack, while leaving air gaps between each layer facilitates rapid moisture transport to and from the spores, and results in a material that can be scaled in two dimensions without compromising hydration/dehydration kinetics (Fig. 1f). Basically, the layered architecture maximizes the surface area for evaporation and condensation and the small thickness of the spore layer (∼3 μm) reduces the travel distance of water within the nanostructured material. Scaling up the size of the material in two dimensions will proportionally increase the surface area for evaporation and condensation, while keeping the thickness of the spore layer constant. Note that the particular wavy design in Fig. 1f also has the advantage of enhancing the effective actuation strain.

We used two complementary strategies to expose hierarchically designed hygroscopic materials to rapidly changing relative humidity. We will discuss them in detail in the following sections. Briefly, the first strategy we used was to control the evaporation rate using a shutter mechanism (Fig. 1g). We will show that coupling the expansion and contraction of the spore-coated films to the shutters results in a self-starting oscillatory movement with net power output. The photo in Fig. 1h shows a fully assembled device that exhibit oscillations when placed above water (see Supplementary Movie 1). The second strategy we used was to move spores in and out of the humid zone. This can be achieved by coupling hydration-induced movements to a rotational motion. For example, gradients in relative humidity near the evaporating surface can induce different degrees of curvature in spore-coated films assembled around a freely rotating disk (Fig. 1i). We will show that the horizontal shift in the centre of mass of the entire structure creates torque that sustains the rotational motion. The photo in Fig. 1j depicts one such device that exhibits continuous rotation when the paper lining the inner surfaces of the device, shown in white, is wetted (see Supplementary Movie 2).

### Hygroscopy-driven artificial muscles

**Figure 2:**
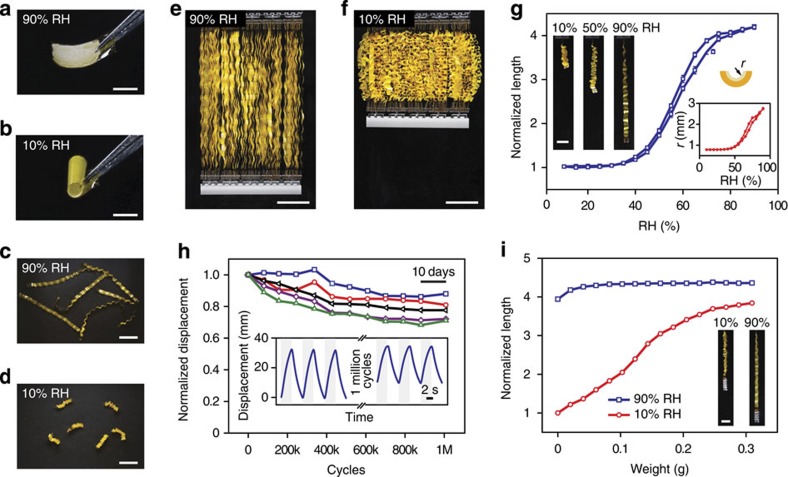
Hygroscopy-driven artificial muscles. Photos of spore-coated polyimide tapes at high (**a**) and low (**b**) relative humidity. Patterning equally spaced spore layers on both sides of a plastic tape creates linearly expanding and contracting muscles (**c**,**d**). HYDRA strips can work in parallel to lift weights (**e**,**f**). (**g**) Elongation of individual strips as a function of relative humidity shows that HYDRAs can quadruple their length. The inset shows estimated radius of curvature of the arcs forming the HYDRAs. Markers indicate the average data values with error bars showing the s.d. calculated from five measurements. (**h**) The elongation of HYDRAs reduced only slightly after one million cycles. The inset shows the time trace of a muscle displacement before and after 1 million cycles. (**i**) The normalized length of a HYDRA strip in dry and humid conditions as a function of load weights. Scale bars, 2 mm (**a**,**b**); 2 cm (**c**–**f**) and 1 cm (**g**,**i**).

Figure 2a,b shows photos of an 8 μm-thick polyimide tape coated with an ∼3 μm-thick spore layer (using *Bacillus subtilis* spores missing most of their outer protein protective layers, due to mutations in *cotE* and *gerE*^27^) changing its curvature in humid and dry conditions. Using the design strategy outlined in Fig. 1c–f, we created longer tapes and assembled them in parallel (see Methods and Supplementary Fig. 1 for the details of sample preparation process). Figure 2c,d shows the resulting dramatic changes in the overall length of tapes in humid and dry conditions. When assembled in parallel, these tapes can lift weight against gravity in dry conditions (Fig. 2e,f and Supplementary Movie 3). Due to their hygroscopy-driven response, we refer to these actuators as hygroscopy-driven artificial muscles, or HYDRAs.

We characterized the actuation behaviour of individual HYDRA strips by placing them inside transparent plastic tubes and flowing air with changing relative humidity (see Methods). Figure 2g shows that HYDRAs can quadruple their length as the relative humidity changes from <30% to >80%, with little hysteresis in the response curve. The amount of moisture absorbed by the HYDRAs in this process is <5% by weight (measured from the differences in strip weights at low and high relative humidity). The average curvature of individual arcs, estimated from the overall lengths of strips, vary from 0.7 mm to 2.8 mm. Note that the response curve in Fig. 2g exhibits an ‘S'-like shape. The response is saturated at high RH, because as the radius of curvature of each arc increases, the end-to-end distances of arcs converge to the contour length of the strip. At the lower RH region, spores approach their fully dehydrated size and this effect limits the degree of contraction observed in the HYDRA strip.

Tests of the stability of actuation performance showed that the extension of strips reduces only slightly even after 1 million cycles and 80 days (Fig. 2h). The strips showed a similar degree of performance reduction when they were subjected to a load weight of 0.1 g (Supplementary Fig. 3). Studies of the mechanism of the observed performance loss would be important as they may allow determining whether the performance can be recovered (Supplementary Note 1 and Supplementary Fig. 2). The time-dependent displacement data given in the inset of Fig. 2h show that the HYDRA strips can respond to changes in relative humidity within ∼3 sec. This is sufficiently fast for practical applications, as we demonstrate later in this work.

When loaded with increasing weight, the range of motion reduces, but remains significant even at load weights that are 50 times more than the strips (0.31 g versus ∼6 mg). Using the force–distance measurements in Fig. 2i, we estimated the work density of the entire strip (by dividing the area enclosed by the curves in Fig. 2i to the weight of the strip), to be ∼17 J kg^−1^, which is close to mammalian skeletal muscles^28^, but comes with extremely large strain. Taken together, HYDRAs require very little moisture for activation and they can generate strains and work densities that can facilitate development of evaporation-driven engines. Note that these characteristics may permit HYDRA applications beyond evaporation-driven engines, as artificial muscles^28^ and bio-hybrid cell-based actuators^29^ for soft robotic systems^30^.

### The oscillatory engine

**Figure 3:**
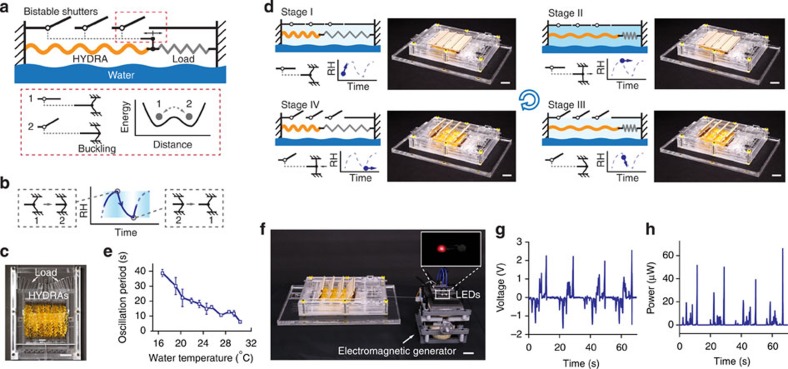
The evaporation-driven oscillatory engine. (**a**) The oscillator comprises horizontally placed HYDRAs coupled to a load spring and shutters that control permeation of moisture. Shutters are connected to a beam that is compressed beyond its buckling limit so that it has two stable configurations. (**b**) As the beam switches its position due to the force exerted by HYDRAs, the shutters open and close and alter the relative humidity of the chamber. (**c**) Picture of HYDRAs assembled in parallel pulling onto load springs. (**d**) Four stages of the oscillatory motion: (Stage I) When the shutters are closed, the relative humidity of the chamber increases, causing HYDRAs to expand. (Stage II) As HYDRAs expand towards the right, they force the buckled beam to switch its position. (Stage III) Opening of the shutters let the relative humidity of the chamber recede, causing HYDRAs to contract. The cycle is completed when contracting HYDRAs pull the buckled beam and force it to switch back to the left configuration (stage IV), which then closes the shutters and brings the system back to stage I. (**e**) Average period of oscillations as a function of water surface temperature. Markers indicate the average data values with error bars showing the s.d. calculated from three measurements. (**f**) Picture of the oscillator connected to an electromagnetic generator. The inset photo of the LEDs is taken during the operation. (**g**,**h**) Voltage and power measured across a load resistor of 100 kΩ. Scale bar, 2 cm.

Using HYDRAs, we have created oscillatory devices that allow and block evaporation in a cyclical fashion, so that energy can be continuously extracted from evaporation. While hygroscopic materials can exhibit spontaneous oscillatory motion due to turbulence or geometric nonlinearities^12^,^15^, these movements are difficult to control and use in devices. To induce oscillations deliberately, we took advantage of the oscillator designs commonly used in electrical circuits. One particularly robust design is a relaxation oscillator that relies on a bistable circuit element (for example, a Schmitt trigger^31^) that is held under feedback control. The relaxation oscillator circuit repeatedly switches between two internal states. The basic architecture of our hygro-mechanical oscillator is designed in analogy with this relaxation oscillator. Figure 3a–d describes how HYDRAs coupled to a buckling beam and a shutter mechanism form a relaxation oscillator.

In our system, we placed HYDRAs horizontally above the water surface and coupled them to a beam that is compressed beyond the buckling limit (bistable element). A load spring holds the HYDRAs under tension (Fig. 3c). The buckled beam controls a shutter mechanism that allows or blocks passage of moist air (feedback) as illustrated in Fig. 3b (see Methods and Supplementary Fig. 4 for details of device fabrication and characterization). This device exhibited oscillations with four key stages (Fig. 3d). As seen in Supplementary Movie 1, this oscillator was self-starting and it generated a piston-like motion.

Figure 3e shows the average period of oscillations as a function of water surface temperature (controlled by a heater placed below the water container). We maintained the temperature and relative humidity, and flow rate of the surrounding air at 25 °C, 20% and 0.6 m s^−1^, respectively. The data show that the increasing of water surface temperature shortened the period of oscillation (Fig. 3e). The device exhibited oscillations even with a water surface temperature well below the air temperature (16 °C versus 25 °C). The most rapid oscillations, observed at 31 °C, had a period of ∼6 s. (We did not record measurements beyond this temperature to prevent condensation). This trend in Fig. 3e can be explained by the temperature dependence of the vapour pressure at the water surface, which affects the evaporation rate significantly. The period of oscillations observed with this oscillator shows that despite the slow natural variations in relative humidity, it is possible to create rapid motions by actively modulating evaporation rates at surfaces.

The rapid piston-like motion of the oscillator allows it to act as an engine, supplying power to external systems. To demonstrate this, we coupled the oscillatory engine to a generator (Fig. 3f) and supplied electricity to light-emitting diodes (LEDs). Water temperature was maintained at 30 °C (see Methods). Depending on the direction of the oscillatory motion, two oppositely connected LEDs gave light repeatedly and in alternating order (see Supplementary Movie 4). We then replaced the LED pair with a resistor and measured the electrical power (Fig. 3g,h). The time course of the voltage across the 100 kΩ resistor and the corresponding power showed that energy was supplied in bursts at power levels reaching 60 μW. Waiting periods that separate these bursts bring the average power to 1.8 μW. These waiting periods could potentially be reduced with more compact designs so that humidity levels change more rapidly. Nevertheless, the ability to generate light with LEDs and the microwatt scale power measured with a load resistor is still significant, especially when the small area of water covered by HYDRAs (9.6 cm × 7.6 cm) is considered.

### The rotary engine

**Figure 4:**
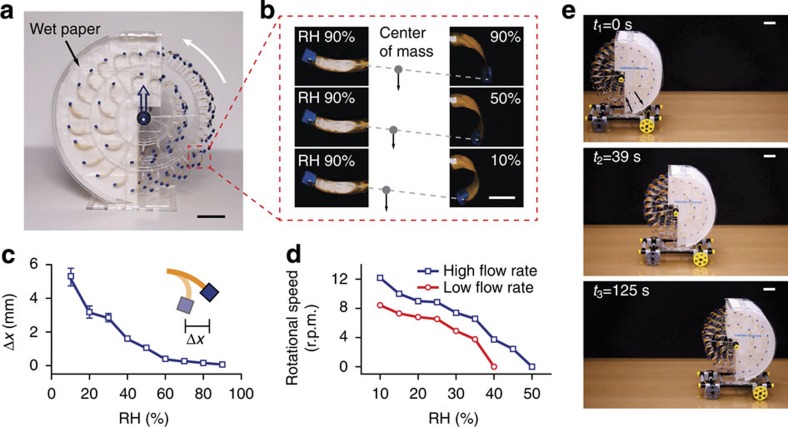
The rotary engine. (**a**) Side view of a rotary engine. Wet paper provides the humidity gradient. Blue plastic blocks weighing 15 mg attached to HYDRAs increase the amount of mass shifting position relative to the axis of rotation. (**b**) Photos and (**c**) measurements showing the horizontal shifts in the positions of plastic blocks attached to HYDRAs. Markers indicate the average data values with error bars showing the s.d. calculated from measurements on five samples. (**d**) Rotation speed measured as a function of external relative humidity and at two different airflow speeds near the device. The rotary engine can drive a vehicle forward if its rotation is coupled to the wheels. (**e**) Snapshots showing the position of a miniature car driven by a rotary engine (see Supplementary Movie 5). Scale bars, 2 cm (**a**,**e**); 5 mm (**b**).

Although the oscillatory engine described so far meets the goal of a scalable device, many devices, particularly those that are used for locomotion, prefer rotary motion. Using the design concept in Fig. 1i, we developed a rotary engine (moisture mill) by assembling HYDRAs around two concentric rings, laser-cut from acrylic glass. Four or five such structures were connected in parallel via a central axis. The entire structure rotates freely around ball bearings (Methods and Supplementary Fig. 5 describe device assembly and characterization in more detail). As seen in Fig. 1j and Fig. 4a, the structure was inserted half way into an enclosure such that the HYDRAs face walls lined with paper (we left out the outermost wall to allow the HYDRAs to be viewed). To increase the torque, we attached small blocks of acrylic at the HYDRAs free ends. Photos (Fig. 4b) and measurements (Fig. 4c) show that the horizontal displacement of the acrylic block was as much as 5.5 mm when the relative humidity on the right side was reduced relative to the left side. As a result of this, the structure began to rotate when the paper was wetted (Supplementary Movie 2). Measurements in Fig. 4d show that the rotation speed depends on the relative humidity outside the chamber and the speed of airflow near the device. The increase in rotation speed with flow rate can be explained by the more efficient mixing with the surrounding air. We note that an airflow specifically directed at HYDRAs can induce torque to rotate the structure. In our case, this effect was negligible, since the data show that beyond a certain relative humidity, the rotations stop despite the presence of airflow.

To demonstrate the application of the rotary engine in locomotion, we created a miniature car by placing the engine above a frame attached to two pairs of wheels, and coupling the engine's rotation to the front wheels with a rubber belt. As the water evaporates from the wet paper, the engine pushes the 0.1 kg car forward (see Fig. 4e and Supplementary Movie 5).

## Discussion

In summary, we demonstrate strategies to scale up a nanoscale energy conversion mechanism to create macroscopic devices. Many interesting nanoscale phenomena benefit from increased surface to volume ratios at small-length scales. However, this property also comes at a price due to slow kinetics that begin to dominate as one tries to scale up the sizes of the structures. Our results show that this challenge can be mitigated in the case of hydration-driven systems and our findings may also be applicable to other systems driven by chemical stimuli. In addition, from a technological standpoint, the demonstrations made here with the evaporation-driven car and the powering of LEDs highlight the so-far overlooked capability of water in the environment to supply useful levels of power. Due to the ubiquity of evaporation in nature and the low cost of materials involved (plastic tapes, hygroscopic materials), the engines presented here may find applications as energy sources for a wide range of off-the-grid systems that function in the environment.

## Methods

### Preparation of HYDRA samples

The preparation process of small HYDRA samples (Fig. 2a,b) starts by cutting 8-μm-thick polyimide sheets (SPEX) into square pieces (4 mm × 4 mm). 0.8 μl Poly-L-lysine solution (0.1 w/v ratio, Sigma-Aldrich) was applied to the top surfaces of the samples using a micropipette and allowed to dry in an environment at an RH of ∼40%. A spore suspension (*cotE gerE* mutant of *Bacillus subtilis*) was mixed with a solution of multipurpose glue (Elmer's Products; ∼2.4 × 10^9^ spores for each 1 μl of glue) using a vortex mixer. The mixture containing ∼4.8 × 10^7^ spores was applied to the top surfaces of the Poly-L-lysine-treated polyimide sheets using a micropipette and allowed to dry in RH of ∼40%. Because spores were assembled on polyimide substrates at their fully hydrated state, these samples presented curvature once the mixture applied on the top surface dried out. The amount of spores formed an ∼3-μm-thick spore layer on top of polyimide substrates as judged by the weight of the spore layer (the density of the spores were assumed to be 1.50 g cm^−3^; ref. 32).

The preparation process of the longer HYDRAs with wavy shapes (Fig. 2c–f) is illustrated in Supplementary Fig. 1. The process starts by deposition of poly-L-lysine on alternating sides of 4 mm × 96 mm polyimide tapes leaving equal spaces between each deposited region (Supplementary Fig. 1a–c). The spore–glue mixture was applied on the areas coated with poly-L-lysine, first on one side (Supplementary Fig. 1d). The mixture was allowed to dry in a humid condition (∼90% RH) to prevent curvature of individual regions before these longer HYDRAs are fully prepared. Then the tape is flipped over and the same amount of the mixture was deposited on the other side (Supplementary Fig. 1e), giving the tapes their wavy shape (Supplementary Fig. 1f).

### Characterization of HYDRA strips

Individual HYDRAs were attached to a piece of acrylic glass and then vertically placed in a transparent glass tube (McMaster-Carr). The RH was monitored by placing a humidity sensor (HIH-4021, Honeywell) near the HYDRAs and recorded to a computer. A stream of air was passed through the glass tube to vary the RH. Control of the RH was achieved by mixing dry air (∼5% RH) provided by a laboratory air source with humid air (∼90% RH) generated by passing the laboratory air through a bubbler (An airstone from JW Pet Company was used to bubble air into water in an Erlenmeyer flask). By controlling the flow rates of dry and humid air sources, the RH inside the glass tube was varied from ∼10% to ∼90%. The lengths of the HYDRA strips were measured with a digital camera. Curvatures of individual arcs (inset of Fig. 2g) were estimated by assuming that each arc has the same shape.

The amount of water absorbed by HYDRA strips were measured from the changes in the total weight of 50 HYDRA strips at 10% and 90% RH. Note that independent of the HYDRA samples, the scale gives different readings at high RH and low RH, presumably due to increased air pressure at high RH. This component is separately measured and subtracted from the measurements of HYDRA strips.

The stability of actuation performance of HYDRAs was measured by alternating RH in the glass tube at a frequency of 0.15 Hz. A solenoid valve (SMC) and a power MOSFET (NTD4960N) was used to switch the air from dry to humid by supplying a square wave using a function generator (Siglent SDG1020). Photos taken by a digital camera monitored the extension of the HYDRA.

To estimate the forces generated by HYDRAs, we tested the motion of HYDRAs with varying load attached when RH is alternated between ∼10% and ∼90%. Masses, made of acrylic glasses, were increased during each trial from 0 g to 0.3 g in increments of 0.02 g. The work density was estimated from the area enclosed by the displacement curves in dry and humid conditions.

### Oscillatory engine assembly

The oscillatory engine is primarily made of stacked layers of laser-cut, 2-mm-thick, acrylic glasses (McMaster-Carr, TAP Plastics). The layers are held together by plastic bars going through the layers (plastic LEGO rods). The bottom components are floating structures that rest on the surface of water and are free to move horizontally. These floating layers provide support for HYDRAs and the bistable structure above (Supplementary Fig. 4a). These structures are made of 1-mm-thick Depron sheets (RCFOAM) and 0.8-mm-thick balsa wood (Dick Blick Art Materials) cut with a laser cutting system (Epilog Helix Laser). The protection layer, which is coated by a hydrophobic material (Hydrobead), allows water vapour to permeate up and meanwhile protect HYDRAs from wetting (Supplementary Fig. 4b). Above the protection layer is the frame (another acrylic layer) that both provides physical support for HYDRAs and connects them to internal or external load (Supplementary Fig. 4c). Eighty-four HYDRA filaments, which cover a surface area of ∼73 cm^2^ (96 mm in length × 76 mm in width), were assembled on the support layer (Supplementary Fig. 4d). A balsa bar was attached to the mobile end of HYDRAs to communicate with top layer controlling the shutter positions. The shutters are fabricated by gluing (Elmer's Products) laser-cut balsa wood and acrylic pieces together. The acrylic pieces on one end of balsa wood surfaces are used to balance the weight of the shutters. After attaching four individual shutter pieces on an acrylic substrate using 13 μm polyimide tapes (CAPLINQ) that served as flexible joints, the shutter layer was placed on the top of HYDRAs with a gap of ∼8 mm (Supplementary Fig. 4e). The oscillatory engine is finished by assembling the final acrylic layer that consists of a bistable beam structure that can be adjusted using two screws placed at both ends of this structure (Supplementary Fig. 4f). This final layer advances and retracts as the bistable beam switches its position. The shutters are coupled to this layer with two threads pulling in opposite directions. Once the rotating disks are inserted into the humid enclosure containing wet papers, the rotary engine starts and runs autonomously as long as papers remain wet.

### Rotary engine assembly

Individual HYDRAs for rotary engine were prepared from 25-μm-thick polyimide tapes (CAPLINQ) that were cut into 6 mm × 14 mm pieces. To increase the torque, blocks of acrylic with a weight of ∼0.015 g were attached to each HYDRAs using multipurpose glue (Elmer's Products). As shown in Supplementary Fig. 5a,b, the HYDRAs were assembled on acrylic rings, which were cut from 1.5-mm-thick acrylic glasses (McMaster-Carr). Individual disks were placed on a stainless steel shaft (1.98 mm in diameter, McMaster-Carr) along with 2-mm-thick acrylic spacers. The shaft is supported by two ball bearings (VXB) that are assembled on an acrylic base (Supplementary Fig. 5b). The humid enclosure consists of cellulose chromatography papers (Sigma-Aldrich) attached to a support made of 0.8 mm and 2 mm thick acrylic glasses (Supplementary Fig. 5c,d). Once the rotating disks are inserted into the humid enclosure containing wet papers, the rotary engine starts and runs autonomously as long as papers remain wet.

### Characterization of the oscillatory engine

The experiment was carried out in an environmental room where the RH and temperature was maintained at 20% and 25 °C, respectively. A water container made of acrylic glasses was placed on a ceramic top heater (Corning, PC-600D). The temperature of water was monitored with a digital thermometer (Deltatrak). The flow rate of the surrounding air was maintained at ∼0.6 m s^−1^ using a computer fan (Sunon) and a hot wire anemometer (Cole-Palmer, EW-30005-85) was used to measure the air velocity. The oscillatory engine was placed on top of the water surface inside the container. While the water temperature was varied from 16 °C to 31 °C, the oscillation frequency of shutters was recorded using a digital camera (Canon EOS Rebel T5i). During the experiment, the compression applied to the buckling beam was slightly adjusted to achieve the maximum oscillation rate for different water temperatures.

### Characterization of the rotary engine

The five-disk rotary engine was placed in a closed chamber of 2-mm-thick acrylic glasses. A stream of humidity-controlled air was pumped into the chamber through plastic tubing. RH and temperature were measured both in the humid enclose of the rotary engine and in the surrounding environment. Humidity and temperature in the chamber was measured using Vaisala HMP77 Humidity and Temperature Probe. Humidity inside the humid enclosure was measured using Honeywell HIH-4021 along with a data acquisition card (NI-USB-6008). The temperature of the wet chromatography paper was measured using a digital thermometer (Deltatrak). A small electric fan was placed in the chamber to control airflow surrounding the rotary engine and the air velocity was monitored using a hot wire anemometer (Cole-Palmer, EW-30005-85). The rotational speed was recorded using a digital camera (Canon EOS Rebel T5i). During the experiment, the chromatography paper was fully saturated with water.

### Experimental set-up for electricity generation

The experiment was carried out in an environmental room where the RH and temperature was maintained at 20% and 25 °C, respectively, The speed of the airflow was maintained at ∼0.3 m s^−1^ (measured with a hot wire anemometer, Cole-Palmer, EW-30005-85). The oscillatory engine, along with water container, was placed on top of a ceramic heater (Corning, PC-600D). The water temperature was monitored using a digital thermometer and kept at 30 °C. We disconnected the internal spring load and connected the HYDRAs to an electromagnetic generator with a short thread. The electromagnetic generator consisted of a stack of magnets rotating between two copper coils. Copper coils are formed by winding a total of 22K feet of 42-gauge magnet wire (Polytech Coil Winding, Tacoma, WA, USA). The magnet stack was composed of two 2″ × ½″ × ¼″ and two 2″ × ½″ × 1/8″ neodymium magnets (K&J Magnetics). We used silicon nitride ball bearings (VXB) to allow magnets rotate with respect to the coils. Two light-emitting diodes (LEDs, HLMP-K155, Avago Technologies) were oppositely connected to the coils. The oscillatory engine rotated the generator and provided power to light the LEDs repeatedly in alternating order. The video was recorded using a digital camera (Canon EOS 6D) while the ambient light was dimmed. The voltage and power generation was measured by replacing the LEDs with a resistor of 100 kω. The voltage across the resistor was measured using a digital multimeter (34410A, Agilent) controlled by a Labview program.

## 

## Additional information

**How to cite this article:** Chen, X. *et al.* Scaling up nanoscale water-driven energy conversion into evaporation-driven engines and generators. *Nat. Commun.* 6:7346 doi: 10.1038/ncomms8346 (2015).

## Supplementary Material

Supplementary Figures and Note Supplementary Figures 1-5 and Supplementary Note 1

Supplementary Movie 1 Evaporation from the water below the device creates a self-starting oscillatory motion. The temperature of the water being poured into the container is 30 °C. The playback speed is 2X of the real time.

Supplementary Movie 2 Evaporation from the wet paper lining the walls of the enclosure creates a rotary motion. The playback speed is real time.

Supplementary Movie 3 A stream of air is passing over HYDRAs. As the relative humidity of the air is switched from high to low, they lift the acrylic block weighing 0.14 g. The playback speed is 2X of the real time.

Supplementary Movie 4 The oscillatory engine is coupled to an electromagnetic generator. The generator is connected to two light emitting diodes (LEDs). As the water beneath the oscillator evaporates, the LEDs light up in alternating order. The temperature of the water is 30 °C. The playback speed is real time.

Supplementary Movie 5 As the water in the wet paper evaporates, it drives the 0.1 kg weighing car forward. The playback speed is 2X of the real time.
