# Supplementary material for: Scaling up nanoscale water-driven energy conversion into evaporation-driven engines and generators
Source: Nat Commun. 2015 Jun 16;6:7346. doi: 10.1038/ncomms8346 (PMC4490384; doi:10.1038/ncomms8346)
Supplement: Supplementary Figures and Note — Supplementary Figures 1-5 and Supplementary Note 1 [file ncomms8346-s1.pdf]

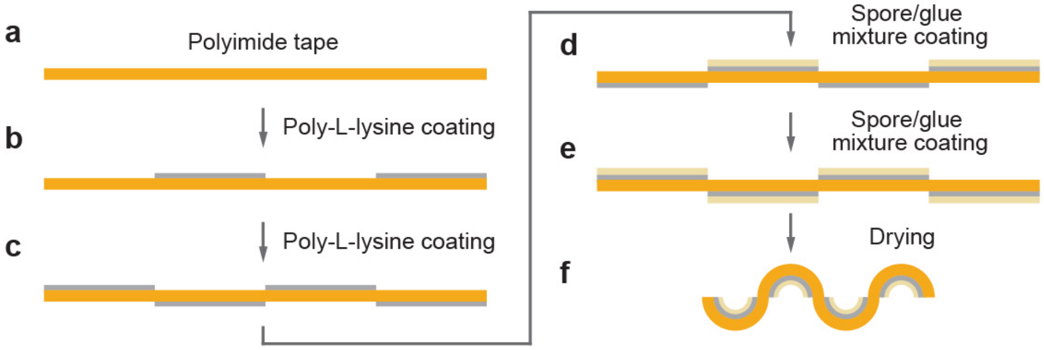

Supplementary Figure 1 | Preparation process of long HYDRAs. (**a**) A polyimide tape served as the substrate for HYDRAs. (**b**, **c**) Poly-L-lysine was periodically applied to the polyimide tape, first on one and then the other. (**d**, **e**) A spore-glue mixture was applied to the poly-L-lysine treated areas, first on one side and then the other. (**f**) The tape gains its wavy shape upon drying.

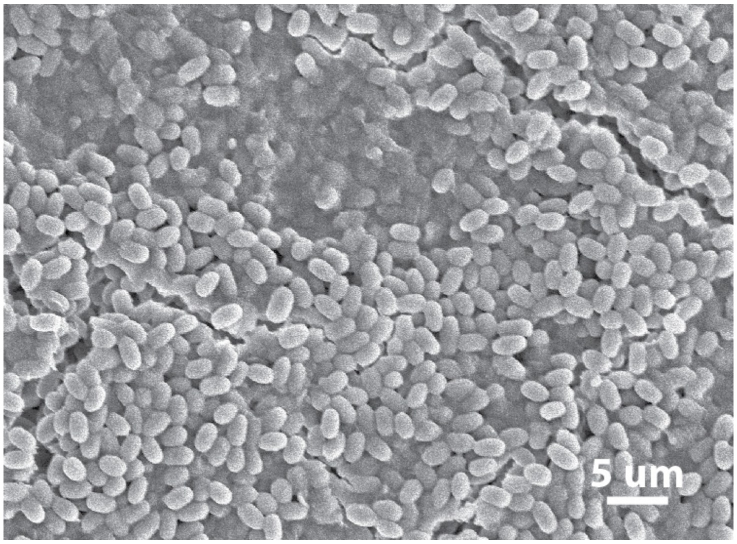

Supplementary Figure 2 | A scanning electron microscopy (SEM) image of the top of a HYDRA sample. Spores assemble into a relatively dense layer upon initial drying of the spore glue mixture.

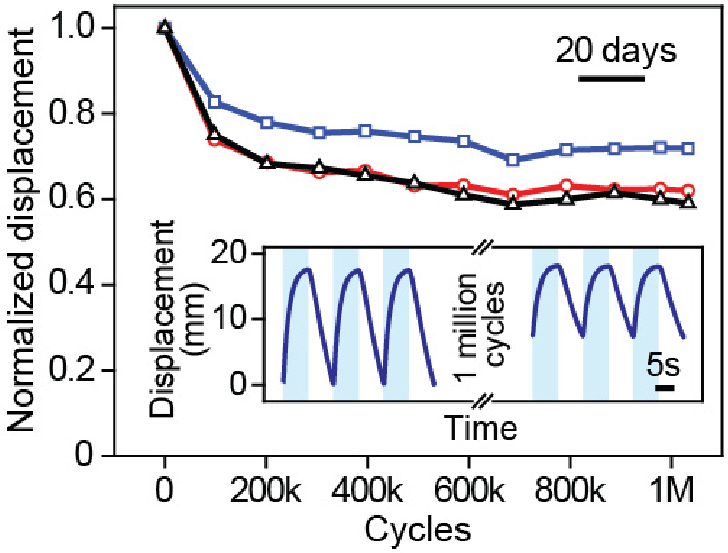

Supplementary Figure 3 | The stability of the actuation performance of HYDRAs. The elongation of HYDRAs lifting 0.1g weights reduced only slightly after one million cycles of alternating high and low RH. The inset shows the time trace of a muscle displacement before and after 1 million cycles.

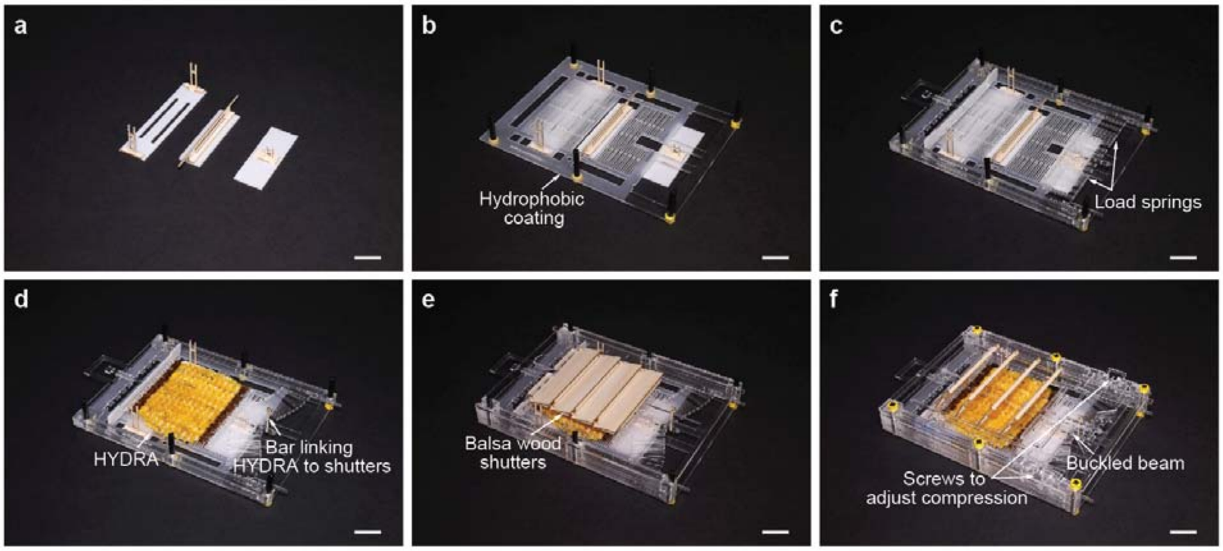

Supplementary Figure 4 | Oscillatory engine assembly. (**a**) Three floating structures support HYDRAs (middle one) and the bistable structure (pieces on the left and right). (**b**) Protection layer keeps HYDRAs away from the water surface but allows moisture to pass through the holes. (**c**) Frame holds HYDRA and the load springs. (**d**) HYDRAs are attached to the frame in (**c**). A vertically oriented bar links the HYDRAs to the shutter layer. (**e**) Shutters placed above HYDRAs control the passage of moisture. (**f**) A frame containing a buckling beam is coupled to the shutters. The vertically oriented bar seen in (**d**) is connected to the buckling beam. Screws on either end of the buckling beam adjust the degree of instability. All scale bars, 20 mm.

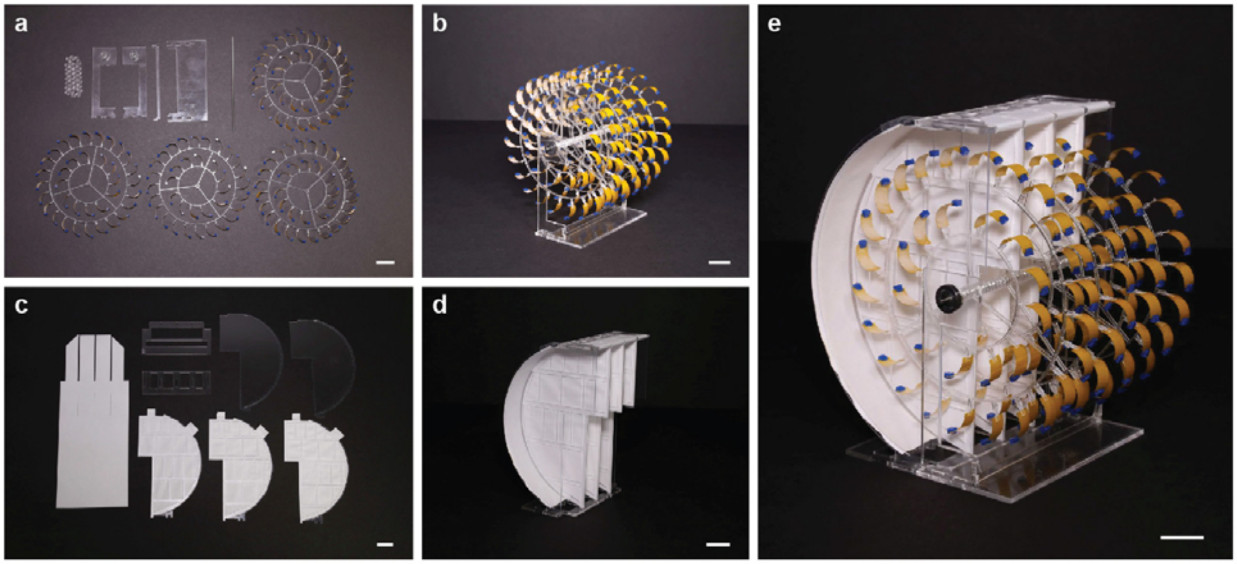

Supplementary Figure 5 | Rotary engine assembly. (**a**, **b**) Rotating disks consist of individual HYDRA pieces and masses affixed to acrylic rings, which were placed on a stainless steel axle that rotates on an acrylic base. Individual components (**a**) and the assembly (**b**). (**c**, **d**) Humid enclosure made of acrylic plates supporting chromatography paper that can be saturated with water. Individual components (**c**) and assembly (**d**). (**e**) Rotating disks are inserted into the humid enclosure so that only half of the HYDRAs are in the humid region at a given time. All scale bars, 20 mm.

Supplementary Note 1

Packing characteristics of spores on HYDRA samples

We examined the surface morphology and packing characteristics of spores on a fully prepared HYDRA sample. Note that other than drying induced aggregation of spores, there is little or no control over spore packing. The SEM image of spores shown in Supplementary Fig. 2 shows that spores were able to assemble into a relatively dense layer on the top of the polyimide substrate. The anisotropic shapes of spores may allow higher packing densities if spore orientations could be controlled, which would increase the energy density of the spore layer. Using the 3-micrometer thickness of the spore layer estimated from Supplementary Fig. 2 and the weight and density of spores on the hydra strips, we estimate the volume fraction of spores to be ~about 60%.
